# Establishment of a Novel Method for Dose Distribution Verification in Stereotactic Radiosurgery (SRS) Using General Semiconductor Array Detectors

**DOI:** 10.7759/cureus.94151

**Published:** 2025-10-08

**Authors:** Shunta Hirano, Takaaki Ito, Yuya Yanagi, Hiroyuki Kosaka, Kenji Matsumoto, Yukinori Matsuo, Hajime Monzen

**Affiliations:** 1 Department of Medical Physics, Graduate School of Medical Sciences, Kindai University, Osaka, JPN; 2 Department of Radiological Technology, Kobe City Nishi-Kobe Medical Center, Kobe, JPN; 3 Department of Radiology, Shiga University of Medical Science, Otsu, JPN; 4 Department of Radiation Oncology, Kindai University Faculty of Medicine, Osaka, JPN

**Keywords:** dose distribution, patient-specific quality assurance, semiconductor dosimeter, stereotactic radiation therapy, vmat-srs

## Abstract

Stereotactic radiosurgery (SRS) involves steep dose gradients and small irradiation fields, which makes ensuring irradiation accuracy extremely important. While general semiconductor array detectors are reusable and cost-effective, their wide detector spacing results in insufficient spatial resolution.

The aim of this study is to establish a novel patient-specific quality assurance (PSQA) method for single-isocenter multiple-target SRS volumetric modulated arc therapy (SIMT-SRS-VMAT) that can evaluate dose distribution with the same accuracy as radiochromic film by virtually narrowing the detector spacing by combining measurement with a general semiconductor array detector and translational movement of the treatment couch.

Single-field plans with a single target and SIMT-SRS-VMAT plans with two targets were created for 1 cm lesions. Measurements using MapCHECK2 (Sun Nuclear, Melbourne, FL, USA) were combined with couch movement to synthesize measurement data at virtual detector spacings of 5, 4, 3, 2, and 1 mm. Root mean square error (RMSE) was used to evaluate profile shape accuracy, and γ analysis was used to evaluate dose distribution agreement.

At virtual detector spacings of 10 mm, 5 mm, 4 mm, 3 mm, 2 mm, 1 mm, and with radiochromic film, the RMSE values in SIMT-SRS-VMAT were 8.02% ± 0.34%, 2.16% ± 0.07%, 1.60% ± 0.08%, 1.48% ± 0.17%, 1.32% ± 0.10%, 1.38% ± 0.03%, and 1.25% ± 0.02%, respectively. The RMSE decreased as the detector spacing narrowed, resulting in profile shapes comparable to those obtained with radiochromic film. In γ analysis, setting the virtual detector spacing to 5 mm enabled analysis covering the entire dose range, and the γ pass rate was comparable to EBT4 (Ashland Inc., Wayne, NJ, USA) for all tolerances.

This study established a novel method of verification of dose distribution with the same accuracy as film, combining measurement with a general semiconductor array detector and translational movement of the treatment couch. With this approach, specialized high-resolution SRS array detectors and disposable radiochromic film are not needed, making SRS PSQA less costly and manpower-intensive.

## Introduction

In high-precision radiotherapy such as stereotactic radiosurgery (SRS), it is recommended to perform patient-specific quality assurance (PSQA) for evaluation of point dose and dose distribution verification to ensure irradiation accuracy [1,2]. In SRS, a high dose is concentrated on a small target, and the dose falls steeply at the outer edge of the target. Typically, prescriptions are made to lower isodose lines, such as 50-80%, to create a conformal and yet intentionally heterogeneous dose distribution with a high central dose and rapid fall-off. These characteristics place stringent demands on the accuracy of dose delivery and its verification [3,4]. Since SRS involves irradiation using many small subfields, the positional accuracy of the multileaf collimator (MLC) has a large impact on the dose distribution [5,6]. Therefore, for SRS PSQA, which requires accurate evaluation of dose distributions with steep gradients in small fields, detectors with low spatial resolution may have insufficient sampling intervals, necessitating detectors with high spatial resolution [7,8], as recommended by recent guidelines, which suggest a distance-to-agreement (DTA) criterion of 1 mm [9].

Two main types of detectors are widely used for SRS dose distribution verification: radiochromic films and semiconductor array detectors. Radiochromic film is considered a gold standard for small-field dosimetry due to its very high spatial resolution and near-tissue equivalence [10]. However, their clinical workflow presents challenges; despite their high cost, they are disposable and require measurement of dose-response curves for each lot. Furthermore, when reading with a scanner, correction for the lateral response effect is required, which is a heavy burden in measurement [11]. On the other hand, detector arrays commonly used for intensity modulated radiation therapy (IMRT) and volumetric modulated arc therapy (VMAT) PSQA may use wide detector spacing (5-10 mm), which is often insufficient to verify the steep dose gradients in SRS [12,13]. To address this, specialized array detectors with improved spatial resolution through smaller detector size and spacing have been developed and are used for SRS verification [14,15].

Recently, single-isocenter multiple-target SRS-VMAT (SIMT-SRS-VMAT) has been used to treat multiple brain tumors [16]. SIMT-SRS-VMAT has enabled the efficient treatment of intracranially scattered targets simultaneously [17]. With array detectors specialized for small irradiation fields, the detection size may not cover the irradiated area in cases where small tumors are scattered [18], forcing users to either select a subsection of the dose distribution for QA or perform multiple measurements, which increases manpower. In addition, the use of radiochromic films and the introduction of array detectors specialized for small irradiation fields in the verification of dose distribution in SIMT-SRS-VMAT incur higher costs and manpower associated with commissioning. The cost-benefit of acquiring a specialized detector depends on the clinical caseload, and facilities with smaller SRS programs may face resource limitations. Therefore, developing a cost-effective verification method that leverages existing equipment is crucial for improving access to high-quality SRS programs.

Several prior studies have explored improving the effective resolution of the diode array detectors by introducing couch or phantom shifts, demonstrating the feasibility of this concept for VMAT QA [19,20]. However, these studies did not directly compare the results with the gold standard of film for complex SRS deliveries. The aim of this study is to establish a novel PSQA method in SIMT-SRS-VMAT that combines measurement with a general semiconductor array detector and translational movement of the treatment couch to generate measurement data with virtually narrower detector spacing and to validate its accuracy against radiochromic film.

## Technical report

Materials and methods

Equipment

**Figure 1:**
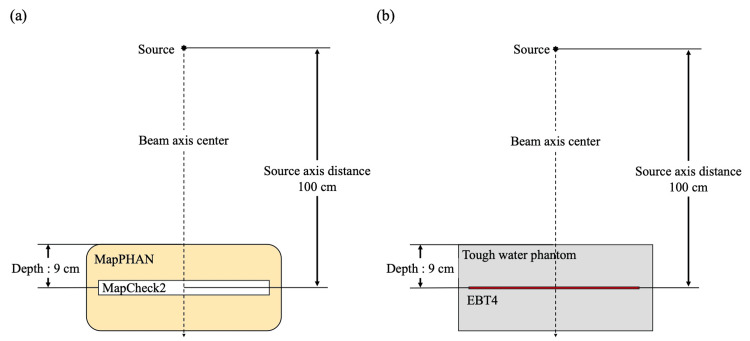
Geometric arrangement of measurement (a) MapCHECK2 measurements (Sun Nuclear, Melbourne, FL, USA); (b) EBT4 measurements (Ashland Inc., Wayne, NJ, USA) Figure was created by the authors of this study.

Treatment planning used the Eclipse treatment planning system (TPS; version 15.6, Varian Medical Systems, Palo Alto, CA, USA). All irradiations used a 6 MV flattening filter-free (FFF) beam (maximum achievable dose rate of 1400 MU/min) on a TrueBeam linear accelerator (Varian Medical Systems, Palo Alto, CA, USA) equipped with standard millennium MLCs. For the measurement, EBT4 (Ashland Inc., Wayne, NJ, USA) was used as the radiochromic film in the center of a Tough water phantom WD (Kyoto Kagaku Co. Ltd., Japan) (PH-40 WD), 18 cm thickness (Figure 1a). MapCHECK2 (Sun Nuclear, Melbourne, FL, USA) with 10 mm detector spacing was inserted into MapPHAN (Sun Nuclear, Melbourne, FL, USA) as a general semiconductor array detector (Figure 1b). For treatment planning, CT images were taken using the Aquilion SP computed tomography (CT) system (Canon Medical Systems, Tochigi, Japan) with a field of view (FOV): 500 mm, slice thickness: 1 mm, and matrix size: 512 × 512 pixels.

Planning

**Figure 2:**
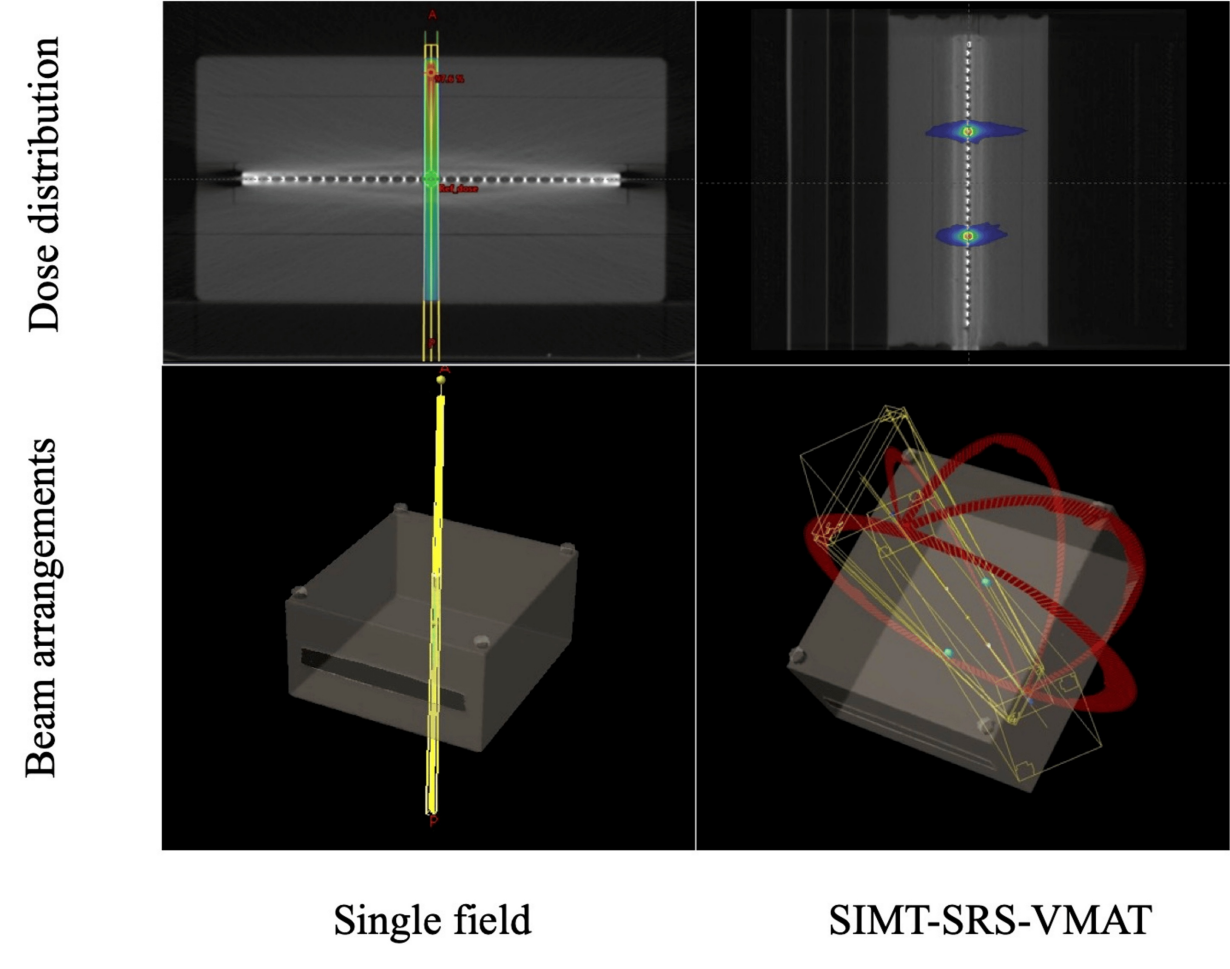
Beam arrangements and dose distribution in single-field irradiation and SIMT-SRS-VMAT plans SIMT-SRS-VMAT: single-isocenter multiple-target stereotactic radiosurgery volumetric modulated arc therapy Figure was created by the authors of this study.

To confirm the feasibility of a validation technique combining measurements using MapCHECK2 and treatment couch movement, a single irradiation plan for a 1 cm target adapted for SRS treatment of brain metastases was created [21,22]. The gantry and collimator angles were set to 0 degrees, and the MLC margin to the planning target volume (PTV) was set to 0 cm. A 200 MU/fr single-field irradiation plan was used to create a steep dose distribution. The Acuros XB algorithm (Version: 15.6, Varian Medical Systems, Palo Alto, CA, USA) was used for dose calculation with dose to medium and a 1 mm grid. A plan with two targets was created for dose distribution validation of the SIMT-SRS-VMAT plan for brain metastases in a clinical setting. Considering the average head size, the targets were offset from the isocenter by ±6 cm in the superior-inferior (SI) direction [23]. The gantry angle, couch angle, and collimator angle were as shown in Table 1 [24]. The D95% prescription for the two PTVs was 20 Gy/fr. Figure 2 shows beam arrangements and dose distribution for single-field irradiation and the SIMT-SRS-VMAT plan that was created.

**Table 1:** Beam arrangement used for SIMT-SRS-VMAT SIMT-SRS-VMAT: single-isocenter multiple-target stereotactic radiosurgery volumetric modulated arc therapy

Plan	Gantry Angle (°)	Couch Angle (°)	Collimator Angle (°)
SIMT-SRS-VMAT	179-181	0	0
	181-0	315	0
	0-179	45	0
	179-0	90	90

Measurement and Creation of Virtual Detector Spacing Data

**Figure 3:**
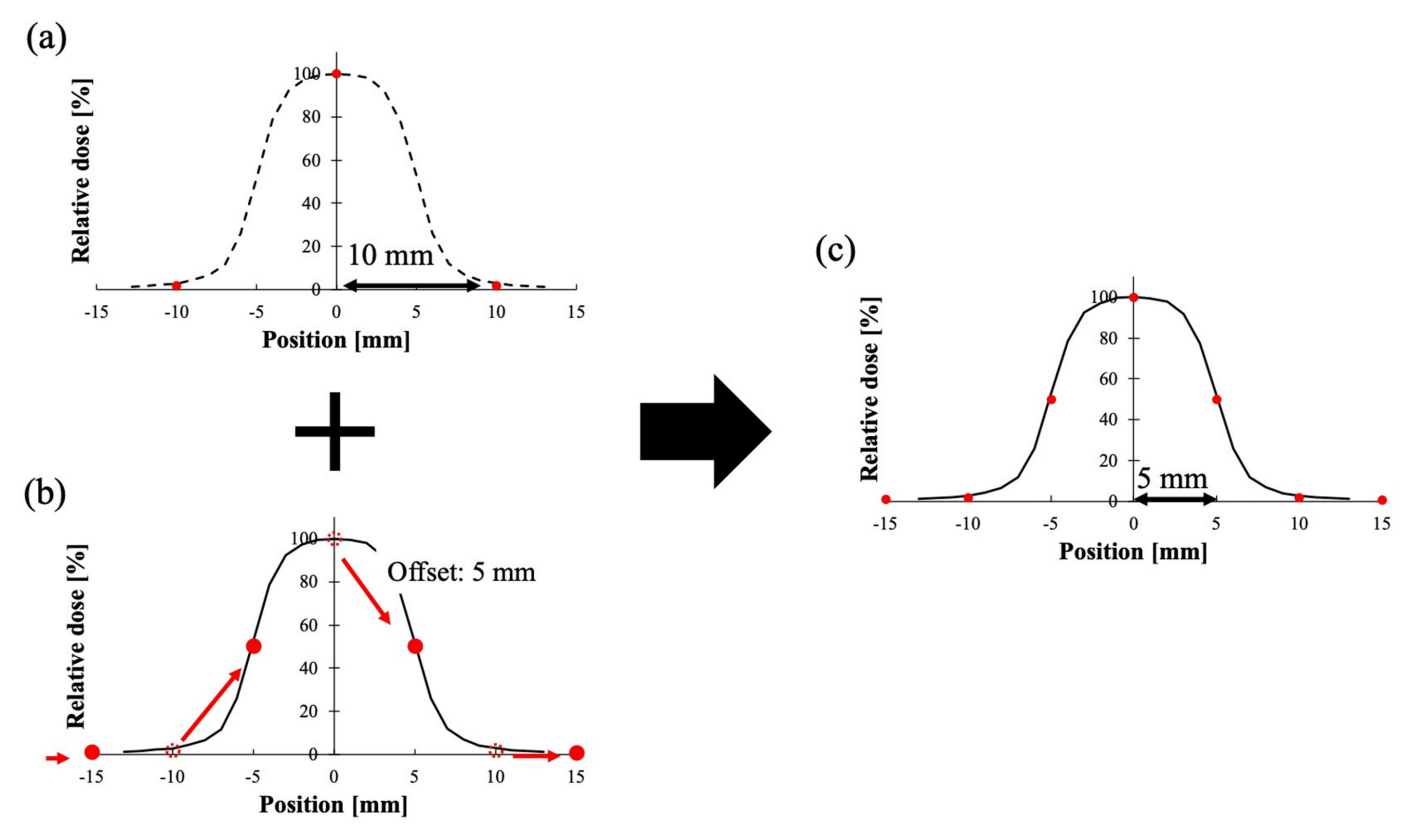
Procedure for creating virtual detector spacing data To create a virtual detector spacing of 5 mm: (a) isocenter measurement data, (b) the 5 mm offset measurement data, (c) the two measurement results are combined to create a virtual 5 mm spacing measurement using MapCHECK2 (Sun Nuclear, Melbourne, FL, USA), which normally has a 10 mm detector spacing. Other detector spacings can also be created by combining multiple offset datasets. Figure was created by the authors of this study.

MapCHECK2 measurements were first performed at the isocenter. To generate data with finer detector spacing, we used manual translational movements of the treatment couch. Specifically, in the SI direction, measurements were taken at the isocenter and at 1-mm increments from 1 to 9 mm (10 positions total). In the right-left (RL) direction, measurements were taken at the isocenter and at a single 5-mm offset. The procedure for generating the virtual detector spacing data is shown in Figure 3. These multiple measurements were then combined to synthesize virtual detector spacing data. A custom Python script (version 3.10.4, Python Software Foundation, Fredericksburg, VA) read the dose data from each measurement file and assigned each dataset to a high-resolution virtual grid according to the known couch shift coordinates. By aligning and merging the shifted datasets, the script produced virtual detector spacings of 5, 4, 3, 2, and 1 mm in the SI direction. In the RL direction, isocenter and 5-mm offset data were combined to yield a virtual detector spacing of 5 mm. For comparison, EBT4 film measurements were performed at the isocenter. Films were stored in the dark for 48 hours after irradiation and scanned with an ES-G11000 scanner (Seiko Epson Corporation, Nagano, Japan) at 72 dpi, 48-bit, red channel [25]. Each MapCHECK2 and EBT4 measurement was repeated three times. MapCHECK2 data were acquired using SNC Patient software (version 6.1, Sun Nuclear, Melbourne, FL, USA), and EBT4 data were analyzed with DD-System (version 14.64, R-Tech Inc., Nagano, Japan).

Evaluation of Required Detector Spacing Using Root Mean Squared Error

The root mean square error (RMSE) was used to evaluate the detector spacing of a general semiconductor array detector that can reproduce a profile shape equivalent to the profile obtained from EBT4 measurement. RMSE represents the difference between two profile shapes, with smaller values indicating greater similarity [26]. The RMSE was calculated from the TPS dose DTPS and the corresponding dose DMeasure at MapCHECK2 and EBT4 using Python in (a). RMSE was calculated at the beam center axis on measurement for MapCHECK2 and virtual detector spacings of 5, 4, 3, 2, and 1 mm. Using the RMSE calculated for the combination of TPS and EBT4 as a reference, evaluated the detector spacing of the semiconductor array detector that provides a profile shape equivalent to that of EBT4. Using the markers attached to the film as a reference, the film and TPS dose distributions were geometrically aligned by matching them to the corresponding coordinates on the TPS.

$\text{RMSE(\%)=}\sqrt{\frac{1}{n}\sum_{i=1}^{n-1}\left( D_{TPS_{i}}-D_{Measure_{i}} \right)^{2}}$・・・(a)

Effect of Different Detector Spacing on γ Pass Rate

The γ analysis was conducted with a virtual detector spacing of 5 mm to reduce the number of measurements for the clinical use of this method. For comparison, calculations were also performed with a virtual detector spacing of 1 mm. To investigate the differences in treatment couch movement direction, the data were measured with a 5 mm offset in the SI or RL direction and evaluated with a virtual detector spacing of 5 mm. Tolerance values of 3%/3 mm, 3%/2 mm, 2%/2 mm, 3%/1 mm, and a threshold of 10% were used for γ analysis.

Results

Evaluation of Required Detector Spacing Using Root Mean Squared Error

**Figure 4:**
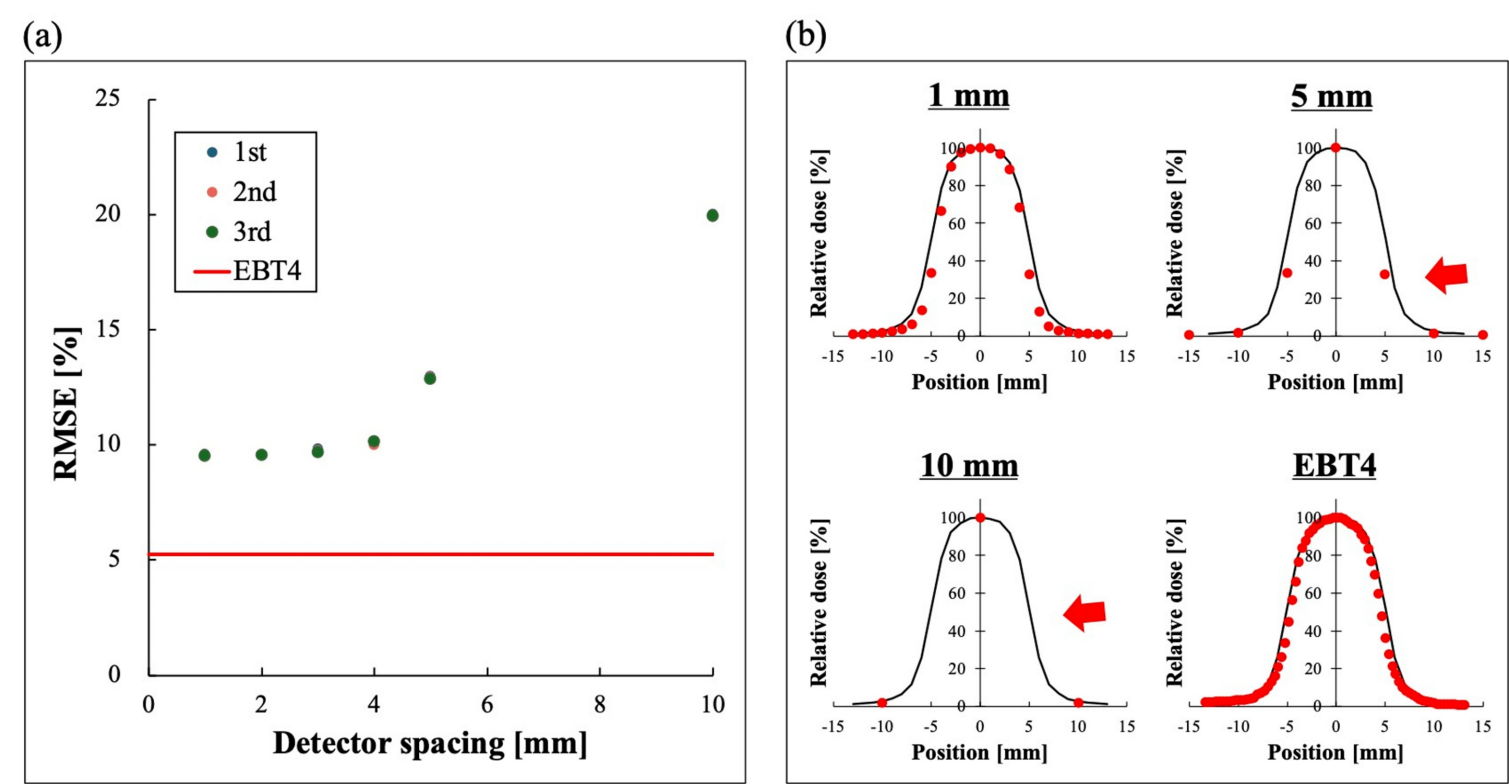
Result of RMSE for single-field irradiation (a) Differences in RMSE for single-field irradiation with 10 mm detector spacing (MapCHECK2), 5, 4, 3, 2, 1 mm virtual detector spacing, and EBT4. Results from three measurements are shown as 1st, 2nd, and 3rd. (b) Profiles calculated by the treatment planning system (black) and doses measured by MapCHECK2 and EBT4 (red dots). Virtually reducing the detector spacing from 10 mm to 5 mm enables the addition of measurement points in the dose gradient region (red arrow). Figure was created by the authors of this study. RMSE: root mean square error

Figure 4a shows the changes in RMSE measured with MapCHECK2 and with virtual detector spacings of 5, 4, 3, 2, and 1 mm for single-field irradiation. By narrowing the virtual detector spacing, the profile error relative to the TPS decreased and approached the RMSE value obtained from the TPS-EBT4 combination. Figure 4b shows the sampling positions for different detector spacings. With a 10 mm detector spacing, measurements are taken at only three points at the maximum dose point and the bottom of the dose gradient, with no measurement points in the dose gradient area. On the other hand, by narrowing the detector spacing, measurement is possible in the dose gradient area, which contributes to the reduction of profile shape error.

**Figure 5:**
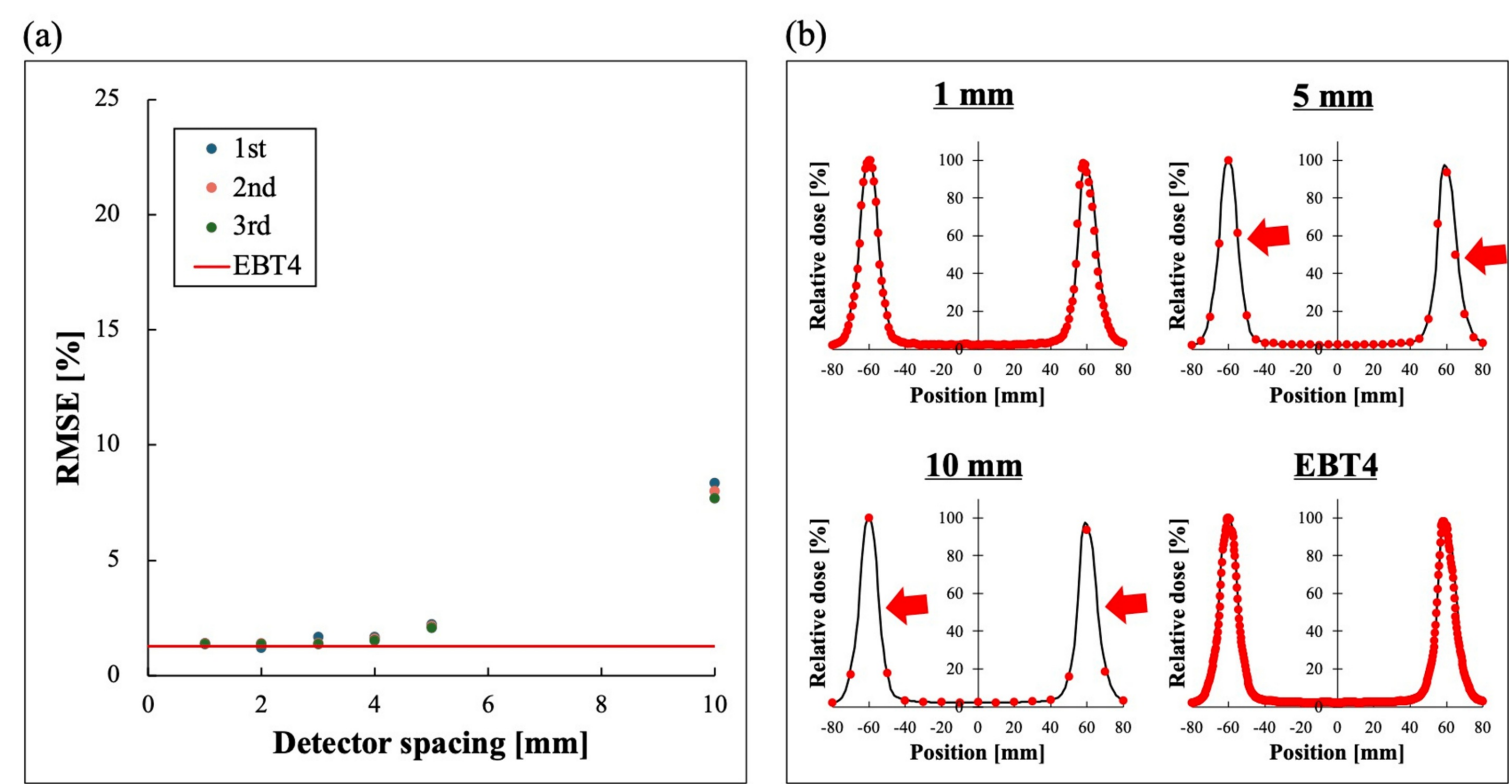
Result of RMSE for SIMT-SRS-VMAT (a) Differences in RMSE for with 10 mm detector spacing (MapCHECK2, Sun Nuclear, Melbourne, FL, USA), 5, 4, 3, 2, 1 mm virtual detector spacing, and EBT4 (Ashland Inc., Wayne, NJ, USA). Results from three measurements are shown as 1st, 2nd, and 3rd. (b) Profiles calculated by the treatment planning system (black) and doses measured by MapCHECK2 and EBT4 (red dots). Virtually reducing the detector spacing from 10 mm to 5 mm enables the addition of measurement points in the dose gradient region (red arrow). Figure was created by the authors of this study. RMSE: root mean square error

The differences in RMSE (Figure 5a) and sampling positions (Figure 5b) in SIMT-SRS-VMAT are shown. In SIMT-SRS-VMAT, as in the case of single-field irradiation, the virtual narrowing of the detector spacing enabled measurement in the dose gradient area and improved the similarity by reducing the error in the profile shape.

Effect of Different Detector Spacing on γ Pass Rate

**Figure 6:**
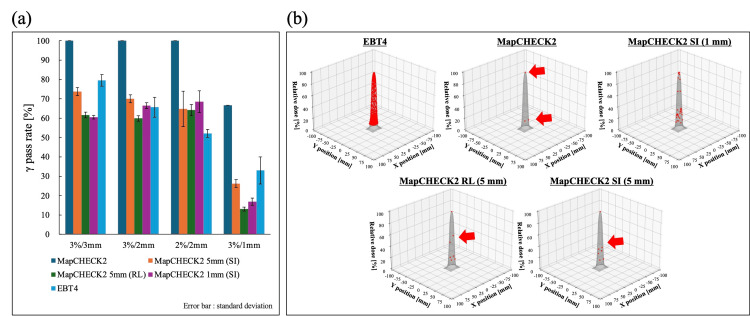
In single-field irradiation, (a) average γ pass rate of three measurements calculated at each tolerance and (b) transition in the measured points due to different detector spacing The gray profile shows the 3D dose profile for single-field irradiation calculated by TPS, and the red plot shows the detector locations used for γ analysis with a threshold > 10%. A 5 mm spacing enables the addition of evaluation points in the medium-dose region (red arrows). Figure was created by the authors of this study. TPS: treatment planning system

In single-field irradiation, the γ-pass rates were calculated for EBT4, MapCHECK2 measurements, virtual detector spacing of 1 mm and virtual detector spacing of 5 mm which was created using 5 mm offset data in the SI and RL directions (Figure 6a). In MapCHECK2 measurements, the average γ pass rates for all three measurements were 100% at the tolerances of 3%/3 mm, 3%/2 mm, and 2%/2 mm, and 66.7% at 3%/1 mm for three measurements. In virtual detector spacing of 1 mm, the average γ pass rates ± standard deviation were 60.5% ± 0.9%, 66.6% ± 1.5%, 68.6% ± 5.6%, 16.8% ± 1.9%. On the other hand, when the virtual detector spacing with 5 mm was 73.8% ± 2.1%, 70.0% ± 2.1%, 64.9% ± 9.2%, and 26.2% ± 2.1% in the SI direction and 61.7% ± 1.4%, 59.9% ± 1.4%, 64.2% ± 2.9%, and 13.1% ± 1.0% in the RL direction. Differences in γ-pass rates were observed depending on the direction of treatment couch movement. The results measured by EBT4 were 79.5% ± 3.0%, 65.7% ± 5.1%, 52.1% ± 2.1%, and 33.0% ± 7.0%, respectively. The locations of the evaluation points used to calculate the γ pass rates are shown in Figure 6b. As in RMSE, with a detector spacing of 10 mm, only three points at the maximum dose point and at the bottom of the dose gradient were available, but with a virtual detector spacing of 5 mm, it was possible to evaluate the γ pass rate including the medium-dose region.

**Figure 7:**
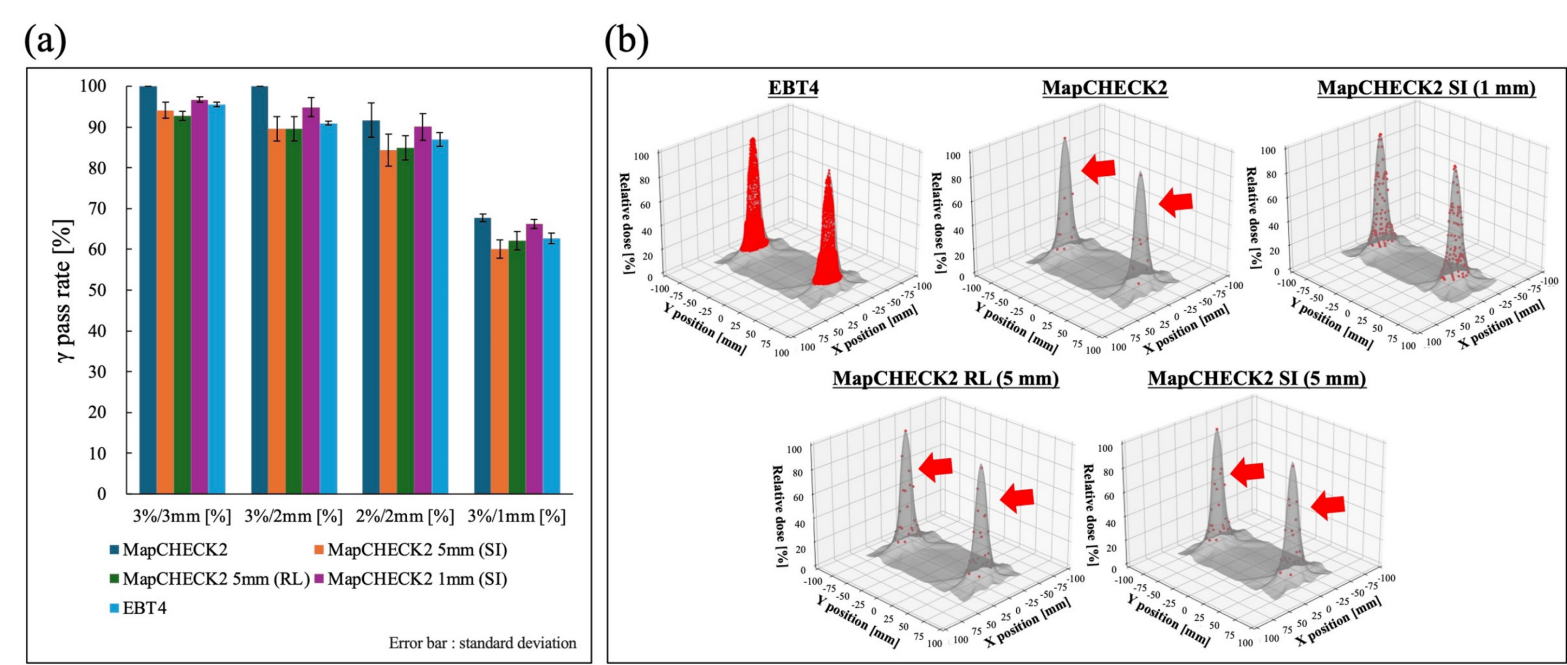
In SIMT-SRS-VMAT, (a) average γ pass rate of three measurements calculated at each tolerance and (b) transition in the measured points due to different detector spacing The gray profile shows the 3D dose profile for single-field irradiation calculated by TPS, and the red plot shows the detector locations used for γ analysis with a threshold > 10%. As in the case of single-field irradiation, 5 mm spacing enables the addition of evaluation points in the medium-dose region (red arrows). Figure was created by the authors of this study. TPS: treatment planning system; SIMT-SRS-VMAT: single-isocenter multiple-target stereotactic radiosurgery volumetric modulated arc therapy

The γ pass rates for SIMT-SRS-VAMT at 3%/3 mm, 3%/2 mm, 2%/2 mm, and 3%/1 mm tolerances are shown in Figure 7a. As in the case of single-field irradiation, MapCHECK2 showed an average γ-pass rate of 100% at the 3%/3 mm and 3%/2 mm tolerances, and 91.7% ± 4.2% at 2%/2 mm, 67.8% ± 1.0% at 3%/1 mm. In virtual detector spacing of 1 mm, the average γ pass rates ± standard deviation were 96.7% ± 0.6%, 94.9% ± 2.4%, 90.1% ± 3.3%, and 66.3% ± 1.1%. On the other hand, when the virtual spacing was 5 mm, the average γ pass rates were 94.1% ± 2.0%, 89.5% ± 3.0%, 84.3% ± 3.9%, and 60.1% ± 2.3% in the SI direction and 92.8% ± 1.1%, 89.5% ± 3.0%, 85.0% ± 3.0%, and 62.1% ± 2.3% in the RL direction. In the SIMT-SRS-VMAT, there was no difference in the γ-pass rate depending on the direction of movement of the treatment couch, which was observed in the case of single-field irradiation. The results measured by EBT4 were 95.5% ± 0.6%, 91.0% ± 0.4%, 87.0% ± 1.7%, and 62.7% ± 1.4%, respectively. By setting the virtual detector spacing to 5 mm, the γ pass rate could be calculated as in EBT4 for all tolerances. The positions of the evaluation points used to calculate the γ pass rate are shown in Figure 7b. With a detector spacing of 10 mm, the evaluation points could not be located between the medium dose region and the maximum dose point, but with a virtual detector spacing of 5 mm, it was possible to evaluate the γ pass rate covering the whole dose range.

## Discussion

In this study, a method combining measurements using general semiconductor array detectors and translational movement of the treatment couch was developed and evaluated for verification of the SIMT-SRS-VMAT with a target size of 1 cm. By virtually reducing the detector spacing, this method improved profile agreement (RMSE) compared to the detector's native 10 mm spacing. Although the RMSE for the finest virtual spacing (1 mm) did not fully match that of the EBT4 film, the improvement was substantial. Furthermore, at a clinically practical virtual spacing of 5 mm, the γ-analysis pass rates were comparable to those of EBT4 film, suggesting that this method is a promising filmless alternative for dose verification.

The results of the RMSE and γ analysis suggest that general semiconductor array detectors can serve as substitutes for radiochromic film when sufficient spatial resolution is achieved through virtual spacing. Previous studies have reported that SRS-dedicated detectors such as SRS MapCHECK (Sun Nuclear, Melbourne, FL, USA, detector spacing: 2.47 mm) and myQA SRS (IBA Dosimetry, Schwarzenbruck, Germany, detector spacing: 0.4 mm) yield verification results comparable to those of radiochromic film. For instance, James et al. demonstrated similar performance between these detectors and film in SRS verification [27]. Sadowski et al. also reported comparable results between SRS MapCHECK and EBT3 film in stereotactic body radiation therapy (SBRT) verification for brain metastases [28]. While these studies support the use of high-resolution semiconductor detectors for filmless verification, the novelty of our study lies in validating a method that uses a general semiconductor array detector with translational movement of the treatment couch and directly comparing its performance against film for SRS PSQA. While other works have investigated resolution enhancement via couch shifts, a direct, comprehensive comparison to film for this specific application is not widely reported.

The effect of couch movement direction on verification accuracy varied with the irradiation technique. This difference is likely due to the anisotropic geometry of the diode units in MapCHECK2, which are elongated in the SI direction (7 × 3 mm²). In single-field irradiation, this geometry may have caused reduced γ pass rates in the RL direction due to partial volume effects. In contrast, SIMT-SRS-VMAT involves both rotational and non-coplanar beams, which likely mitigates the impact of couch movement direction. Therefore, it is considered unnecessary to consider the treatment couch movement direction in the case of a plan using VMAT when conducting verification in SRS using this method.

This method is applicable for verifying dose distributions in multi-target SRS cases with lesions ≥ 1 cm in diameter. In this study, accurate dose profiles were obtained for two 1 cm targets. Clinical reports have documented cases of simultaneous irradiation of three or more brain metastases [29,30]. Given the demonstrated accuracy and spatial resolution at 5 mm virtual spacing, the proposed method may be extended to other multi-target SRS applications, provided that the target size is at least 1 cm.

An additional advantage of using a general semiconductor array detector such as MapCHECK2 lies in its wide measurement area (260 × 320 mm²), which far exceeds that of SRS-specific detectors like SRS MapCHECK (77 × 77 mm²) and myQA SRS (120 × 140 mm²). This enables coverage of the average adult cranial dimensions (172.00 mm lateral, 175.45 mm anterior-posterior) [31], thus facilitating SRS verification involving the whole brain. However, as in SRS-dedicated detectors, a key drawback is that MapCHECK2 is limited to measurements in a single plane. For SIMT cases where targets do not lie on the same coronal or sagittal plane, multiple measurements at different phantom positions would be required, or a clinically representative plane must be chosen for verification.

This study has several limitations that should be considered. Positional and setup uncertainties: The method relies on precise couch movements and setup. Any errors, whether from the couch positioning system or human setup, could propagate into the synthesized data, affecting its accuracy. The impact of such potential errors was not quantified in this study. Limited scope of test cases: The validation was performed using only idealized 1 cm spherical targets and a single two-target SIMT-SRS-VMAT plan. The method's performance on smaller targets (< 1 cm), larger targets, more complex shapes, and more challenging clinical plans (e.g., targets near organs at risk, off-axis targets) has not been evaluated. Dosimetric evaluation metrics: While this study focused on gamma analysis, comparing target center point dose and peak dose is also crucial for SRS quality assurance, making it an important future task.

## Conclusions

This proof-of-concept study demonstrates that combining general semiconductor array detectors with couch translation can achieve dose verification accuracy comparable to that of film for 1-cm SRS targets. These findings highlight the feasibility of this method as a cost-effective alternative for facilities with limited SRS case load. Further validation with larger sample sizes and diverse clinical scenarios is necessary to confirm its generalizability and reliability.

## References

[1] Miften M, Olch A, Mihailidis D (2018). Tolerance limits and methodologies for IMRT measurement-based verification QA: recommendations of AAPM Task Group No. 218. Med Phys.

[2] Pulliam KB, Followill D, Court L, Dong L, Gillin M, Prado K, Kry SF (2014). A six-year review of more than 13,000 patient-specific IMRT QA results from 13 different treatment sites. J Appl Clin Med Phys.

[3] Zhao B, Jin JY, Wen N, Huang Y, Siddiqui MS, Chetty IJ, Ryu S (2014). Prescription to 50-75% isodose line may be optimum for linear accelerator based radiosurgery of cranial lesions. J Radiosurg SBRT.

[4] Walter YA, Dugas JP, Broekhoven BL, Jacobs TD, Han M, Wang CJ, Wu HT (2024). Effect of prescription isodose line on tissue sparing in linear accelerator-based stereotactic radiosurgery treating multiple brain metastases using dynamic conformal arcs. J Appl Clin Med Phys.

[5] Nithiyanantham K, Mani GK, Subramani V, Mueller L, Palaniappan KK, Kataria T (2015). Analysis of direct clinical consequences of MLC positional errors in volumetric-modulated arc therapy using 3D dosimetry system. J Appl Clin Med Phys.

[6] Kim JI, Park SY, Kim HJ, Kim JH, Ye SJ, Park JM (2014). The sensitivity of gamma-index method to the positioning errors of high-definition MLC in patient-specific VMAT QA for SBRT. Radiat Oncol.

[7] Padelli F, Aquino D, Fariselli L, De Martin E (2022). IBA myQA SRS detector for CyberKnife Robotic Radiosurgery quality assurance. Appl Sci.

[8] Rose MS, Tirpak L, Van Casteren K, Zack J, Simon T, Schoenfeld A, Simon W (2020). Multi-institution validation of a new high spatial resolution diode array for SRS and SBRT plan pretreatment quality assurance. Med Phys.

[9] Cirino E, Benedict SH, Dupre PJ (2025). AAPM-RSS Medical Physics Practice Guideline 9.b: SRS-SBRT. J Appl Clin Med Phys.

[10] Das IJ, Francescon P, Moran JM (2021). Report of AAPM Task Group 155: megavoltage photon beam dosimetry in small fields and non-equilibrium conditions. Med Phys.

[11] Niroomand-Rad A, Chiu-Tsao ST, Grams MP (2020). Report of AAPM Task Group 235 Radiochromic Film Dosimetry: an update to TG-55. Med Phys.

[12] Guo J, Zhu M, Zeng W (2024). Multileaf collimator modeling and commissioning for complex radiation treatment plans using 2-dimensional (2D) diode array MapCHECK2. Technol Cancer Res Treat.

[13] Lee YC, Kim Y (2021). A patient-specific QA comparison between 2D and 3D diode arrays for single-lesion SRS and SBRT treatments. J Radiosurg SBRT.

[14] Walter YA, Durham PF, Hubbard AN, Burrell WE, Wu HT (2025). Improving dose delivery in non-coplanar cranial SRS: stereoscopic x-ray-guided mitigation of table walkout errors. J Appl Clin Med Phys.

[15] Junis I, Yousif Y, Stensmyr R, Barber J (2024). Comprehensive characterisation of the IBA myQA SRS for SRS and SBRT patient specific quality assurance. Phys Eng Sci Med.

[16] Uto M, Torizuka D, Mizowaki T (2022). Single isocenter stereotactic irradiation for multiple brain metastases: current situation and prospects. Jpn J Radiol.

[17] McCulloch J, Pawlowski J, Kirby N (2020). Patient-specific dose quality assurance of single-isocenter multiple brain metastasis stereotactic radiosurgery using PTW Octavius 4D. J Appl Clin Med Phys.

[18] Iwai Y, Ozawa S, Ageishi T, Pellegrini R, Yoda K (2014). Feasibility of single-isocenter, multi-arc non-coplanar volumetric modulated arc therapy for multiple brain tumors using a linear accelerator with a 160-leaf multileaf collimator: a phantom study. J Radiat Res.

[19] Kunii Y, Tanabe Y, Higashi A, Nakamoto A, Nishioka K (2023). Effects of high-resolution measurements between different multi-row detectors on volumetric modulated arc therapy patient-specific quality assurance. IJRR.

[20] Keeling VP, Ahmad S, Algan O, Jin H (2014). Dependency of planned dose perturbation (PDP) on the spatial resolution of MapCHECK 2 detectors. J Appl Clin Med Phys.

[21] Roper J, Chanyavanich V, Betzel G, Switchenko J, Dhabaan A (2015). Single-isocenter multiple-target stereotactic radiosurgery: risk of compromised coverage. Int J Radiat Oncol Biol Phys.

[22] Pokhrel D, Palmiero AN, Bernard ME, Clair WS (2021). Dynamic conformal arcs-based single-isocenter VMAT planning technique for radiosurgery of multiple brain metastases. Med Dosim.

[23] Ito T, Kubo K, Monzen H, Yanagi Y, Nakamura K, Sakai Y, Nishimura Y (2023). Overcoming problems caused by offset distance of multiple targets in single-isocenter volumetric modulated arc therapy planning for stereotactic radiosurgery. J Med Phys.

[24] Ohira S, Sagawa T, Ueda Y (2020). Effect of collimator angle on HyperArc stereotactic radiosurgery planning for single and multiple brain metastases. Med Dosim.

[25] Kamomae T, Miyabe Y, Sawada A (2011). Simulation for improvement of system sensitivity of radiochromic film dosimetry with different band-pass filters and scanner light intensities. Radiol Phys Technol.

[26] Jensen PJ, Zhang J, Koontz BF, Wu QJ (2021). A novel machine learning model for dose prediction in prostate volumetric modulated arc therapy using output initialization and optimization priorities. Front Artif Intell.

[27] James S, Al-Basheer A, Elder E (2023). Evaluation of commercial devices for patient specific QA of stereotactic radiotherapy plans. J Appl Clin Med Phys.

[28] Sadowski BM, Fillmann M, Szałkowski D, Kukołowicz P (2022). Evaluation of SRS MapCHECK with StereoPHAN phantom as a new pre-treatment system verification for SBRT plans. PJMPE.

[29] Palmer JD, Sebastian NT, Chu J (2020). Single-isocenter multitarget stereotactic radiosurgery is safe and effective in the treatment of multiple brain metastases. Adv Radiat Oncol.

[30] Lau SK, Zakeri K, Zhao X (2015). Single-isocenter frameless volumetric modulated arc radiosurgery for multiple intracranial metastases. Neurosurgery.

[31] Kuo C-C, Wang M-J, Lu J-M (2020). Developing sizing systems using 3D scanning head anthropometric data. Measurement.

